# On Supporting University Communities in Indoor Wayfinding: An Inclusive Design Approach

**DOI:** 10.3390/s21093134

**Published:** 2021-04-30

**Authors:** Catia Prandi, Giovanni Delnevo, Paola Salomoni, Silvia Mirri

**Affiliations:** 1 Department of Computer Science and Engineering, University of Bologna, 40126 Bologna, Italy; catia.prandi2@unibo.it (C.P.); paola.salomoni@unibo.it (P.S.); silvia.mirri@unibo.it (S.M.); 2 ITI-LARSyS, 9020-105 Funchal, Portugal

**Keywords:** indoor wayfinding, indoor localization technologies, inclusive design, BLE beacon, Internet of Things, people with disabilities

## Abstract

Mobility can be defined as the ability of people to move, live and interact with the space. In this context, indoor mobility, in terms of indoor localization and wayfinding, is a relevant topic due to the challenges it presents, in comparison with outdoor mobility, where GPS is hardly exploited. Knowing how to move in an indoor environment can be crucial for people with disabilities, and in particular for blind users, but it can provide several advantages also to any person who is moving in an unfamiliar place. Following this line of thought, we employed an inclusive by design approach to implement and deploy a system that comprises an Internet of Things infrastructure and an accessible mobile application to provide wayfinding functions, targeting the University community. As a real word case study, we considered the University of Bologna, designing a system able to be deployed in buildings with different configurations and settings, considering also historical buildings. The final system has been evaluated in three different scenarios, considering three different target audiences (18 users in total): i. students with disabilities (i.e., visual and mobility impairments); ii. campus students; and iii. visitors and tourists. Results reveal that all the participants enjoyed the provided functions and the indoor localization strategy was fine enough to provide a good wayfinding experience.

## 1. Introduction

Finding the appropriate position and direction in the environment is an important issue for human beings since a long time ago. In fact, many of the most important human activities are based on the capabilities of orientating and independently moving in the space and in the surrounding environment [1,2].

Its importance is evident even in Greek mythology: Ariadne’s string had a significant role, being a mean that let Theseus retrace his way out of the labyrinth of the Minotaur. Nowadays, modern Ariadne’s strings are supporting people in orientating and in moving within buildings and across urban environments. Basically, they are the result of the exploitation of mobile devices equipped with sensors that receive signals from external sources, employing specific positioning and wayfinding algorithms.

Even though radio waves of the satellites propagate for enormous distances, in the proximity of the user they can encounter obstacles, such as walls, roofs, or vegetation that can inhibit their reception. For this reason, inside buildings, it is unlikely to receive a usable GPS signal to translate the navigation services designed for the outside into enclosed spaces. Therefore, it is necessary, in order to accurately localize individuals in indoor environment and to provide them wayfinding and navigation support, to find alternative technologies [3].

The wide diffusion of smartphones and the emergence of IoT infrastructures and smart objects have been only a small part of the huge technological revolution, that has distinguished the last years and has seen an ever-growing interconnection and interaction between people and mobile devices. The paradigm shift envisioned by the Internet of Things represents a unique opportunity in supporting people, especially those with disabilities, who could get a significant benefit, improving their independence in conducting their daily life activities [4]. For instance, these activities are ranging from letting people with visual and/or mobility impairments control smart home lights, heating, and appliances in an easier way, to managing home security systems; from moving within a complex and unknown building, to get additional information about architectural elements and points of interest, and so on. An IoT focused on users’ and communities’ needs could really play a key role in terms of inclusiveness and accessibility [5].

In this context, taking into account indoor positioning and wayfinding, some questions arise: which are the most suited technologies for user’s positioning in a certain context? Which design approach better lets to meet users and communities’ needs?

In this paper, we aim to answer these questions, by presenting a case study based on the design, development, and evaluation of a pervasive system, named AlmaWhere. The main goal of this work is to study solutions so as to identify how to equip users with a system to navigate an indoor environment, localizing Points of Interest (PoIs). Specific attention has been paid in the design phase, with the aim of providing an inclusive tool, meeting the specific needs of users with disabilities, in particular those people with mobility impairments (who need tailored paths within a building, avoiding architectural barriers, such as stairs and steps) and people with visual impairments (who need specific landmarks, turn by turn instructions, verbose descriptions of the environment, as well as accessible user interfaces and interaction mechanisms). As per other accessibility techniques and solutions, we aimed to get benefits for a larger users’ community, taking advantage of a curb-cut effect [6]. Indoor positioning and wayfinding have been based on the use of BLE (Bluetooth Low Energy) beacon devices, evaluating specific techniques by involving target users. AlmaWhere has been applied as a real case study at the University of Bologna (which is composed of five campuses in five different cities), this allowed us to conduct different field studies, by involving three communities, including students with disabilities and tourists who are interested in visiting the historical building of the most ancient university of the western world. This paper discusses the adopted techniques and design approach and it reports the results we obtained from the field study evaluations, carried out in three different scenarios and with three target groups.

The remainder of this paper is organized as follows. Section 2 presents the main related work, focusing on indoor positioning techniques and on main strategies to support users with disabilities in navigating and wayfinding activities. Section 3 describes the design issues that have driven our work. Section 4 illustrates the system walkthrough, presenting our real case scenario, while Section 5 reports the results of the evaluations we have conducted with target users. Finally, Section 6 concludes the paper by hinting at some future work.

## 2. Related Work

This Section is organized into two parts. In the first one, we present the state-of-the-art of indoor positioning, illustrating the different approaches that have been proposed over the years. The second one, instead, describes some projects devoted to the development of indoor wayfinding/navigation systems for people with disabilities, focusing on similarities and differences with the AlmaWhere project.

### 2.1. Indoor Positioning

The key element of any wayfinding system is its positioning system. While in outdoor environments the Global Positioning System (GPS) is an accurate and standard solution [7], the indoor localization is still an open challenge. Different approaches have been proposed to address this challenge. A first categorization can be done based on whether or not they require additional infrastructures.

Being able to locate users without the need for any particular infrastructure is certainly an attractive prospect, given the obvious advantages regarding the costs (of installation and maintenance) and the scalability of the whole system. The first family of approaches, that falls in this group, is the one based on the real-time processing of images captured by smartphones camera. Essentially, computer vision techniques or deep learning algorithms are employed to detect specific markers in the environment, that allow us to understand the user position [8,9]. In the context of visually impaired users, the idea at the basis of such approaches is to replace the lost sense (i.e., the sight) with an artificial eye [10]. These approaches have the additional advantage of being able to detect obstacles [11]. However, similar approaches also present some major drawbacks. The most relevant is the possible obstruction of the visual field by people and objects. In fact, for the correct functioning of this type of system, it is essential not to have obstacles between the camera and the markers, a constraint hard to satisfy in crowded environments. Another drawback concerns the high battery consumption resulting from the continuous use of the camera and from the real-time processing of the captured images. A variant of the latter approach utilizes visible light communication technology [12,13].

The other localization approach that does not require additional infrastructures is the one based on Wi-Fi signals. It falls in this category since it is possible to assume that the public (and private) buildings are equipped with Wi-Fi. This approach typically exploits the Received Signal Strength Indicator (RSSI) values from multiple access points to determine the users’ position [14,15]. As can be easily deduced, the accuracy of such an approach strongly depends on the number, and the relative position, of the access points. This is an important limitation since the access points deployment is usually performed to guarantee an adequate internet connection and not to ensure precise localization in every place of the building. Moreover, since Wi-Fi signals are unstable and noisy, the received RSSI values in the same place could vary significantly over time, making localization difficult. To partially mitigate these issues, further access points may be added at specific points with the aim of improving the localization accuracy. Another option is to employ sensor fusion techniques to combine data coming from other smartphone sensors to RSSI [16].

With regard to the approaches that require additional infrastructure, the main ones are based on Radio-Frequency Identification (RFID) and beacons. RFID uses radio frequencies to perform wireless communication between a tag and a receiver. RFID tags can be active or passive [17,18]. Passive tags do not carry batteries but are powered by the reader signal. For this reason, the communication range is limited. On the other hand, active tags (equipped with batteries) can transmit at higher power levels and thus have a longer range. Despite this, the interest of the scientific community is primarily on passive tags. In fact, while active RFIDs are just another example of local-area communication technologies, passive tags present unique advantages from a system maintenance perspective: the limited cost per tag and no need for replacement due to low battery. Notwithstanding these benefits, the use of RFID in navigation systems remained limited due to the following factors [19]. First of all, the reader devices must be at a very short distance to be able to detect the passive tag, a clear issue for people with visual impairments [20]. Furthermore, RFID technology requires a specific receiver that is rarely included in smartphones or other commonly used objects [21].

Beacons, instead, are devices that use BLE to broadcast some data at a fixed time interval. They have emerged as one of the most promising technology in the Internet of Things (IoT) ecosystem [22]. Their power supply depends on the constructive choices of the producers. The most adopted solution is to equip them with batteries. Others need to be connected to a power outlet or to a USB port. Obviously, batteries allow an easier deployment but they will have to be cyclically replaced. Beacons can work with different communication protocols: iBeacon [23], AltBeacon [24], and Eddystone [25]. The transmitted data, that vary based on the adopted protocol, can be, for example, a Universally Unique IDentifier (UUID), a 24-character string, or a simple URL. Together with the transmitted data, it is also available the RSSI values. Hence, the same considerations made for Wi-Fi based approaches are valid. Since the information is transmitted via Bluetooth, there is maximum compatibility with smartphones, contrary to what happens with the RFID.

For all the aforementioned reasons, in the AlmaWhere project, we decided to employ BLE beacons. It is worth to notice that, despite the presented advantages, this IoT technology still present open issues that need to be resolved in order to provide a stable, accurate, and scalable localization solution.

### 2.2. Indoor Navigation Systems for People with Disabilities

Different projects were born with the aim of developing an indoor navigation system to support people with disabilities over the years. They are focused on a specific disability like mobility [26,27,28,29] or visual [30,31] or cognitive impairments [32,33]. Analyzing the current literature, it is possible to notice how systems devoted to support visually impaired users are the most studied, employing different approaches like computer vision [34], augmented reality [35,36], RFID [37], and wireless optical communications [38].

Here we provide a description of the more relevant projects that focus on visually impaired users and are based on beacon technology, detailing the chosen approaches, the techniques employed, and the implementation choices made.

NavCog (https://www.cs.cmu.edu/~NavCog/navcog.html Last accessed on 23 February 2020) is probably the most known project devoted to the implementation of a navigation system for visually impaired users [39]. It is based on BLE beacons and exploits the fingerprinting of RSSI values, using Kalman filter and a set of local regression models. This technique requires a distance of about 5–10 m between beacons, that have to be deployed as a grid. A mobile application for iOS is available [31]. The only goal of such an application is to provide turn-by-turn navigation between two points. During navigation, the information can be provided through vocal messages or sonification. The vocal messages speed can be regulated (slow or fast). The map, the POIs (Points of Interest), and the beacons deployment can be updated through a web interface. Another interesting project is StaNavi [40], a navigation system that provides turn-by-turn directions inside the Tokyo Station. It employs BLE beacons too, even if using a different technique: the proximity one. With this technique, the only constraint is to not put beacons densely to avoid conflicts between signals. StaNavi is implemented for the iOS 8 platform, so it runs only on Apple devices. It provides two main functionalities: the Free Roam, which allows getting the current location and detailed information about the surrounding; and the Navigation, thanks to which a user can obtain turn-by-turn directions, from his/her current location to a chosen destination. The path is always computed by the server, that uses Dijkstra’s Shortest Path and sends the result in an XML file. The user interacts with the User Interface (UI) via ad hoc gestures. The indications are read using Text-to-Speech APIs, included from iOS 7. Last but not least, there is GuideBeacon [41]. Similar to StaNavi, it localizes the user thanks to the proximity to BLE beacons. To avoid the conflicts caused by the variability of RSSI signals, they developed an algorithm based on a window of the last n signals. Unlike the previous project, the mobile application is developed for the Android OS. It provides only the navigation module, allowing a user to reach a destination from his/her current position. The map of the building is stored on a server. The interaction between the users and the mobile application takes place through Speech-Recognizer and Text-To-Speech.

Finally, even though it regards an outdoor wayfinding system, it is worth mentioning the work of Fogli, Arenghi, and Gentilin [42]. In fact, in the design phase, they exploit a Universal Design approach with the aim of developing a general system able to fit the needs of everyone to find the best path. This was possible thanks to an interdisciplinary team involving not only HCI experts and developers but also civil engineers, architects, and representatives of various communities, including that of people with disabilities.

As stated at the beginning of this Section, the AlmaWhere project presents some similarities, with the above-described projects but it also differs in various aspects. Considering the similarities, it is based on BLE beacons and it employs the proximity technique to identify the location of users inside the building and it uses the compass to provide accurate turn-by-turn indications. Regarding the differences, we can identify at least four main ones. First of all, it has been designed following an inclusive design approach, aimed to support not only visually impaired users, but also people with disability in general (i.e., mobility impairments) and, more in general, any person interested to explore the indoor environment (e.g., first-time visitors) [43]. AlmaWhere has been developed for both iOS and Android devices. Moreover, AlmaWhere provides two functionalities in addition to the navigation module, the Around You and the Have a Tour ones (as described in the following Sections). Finally, the interaction between users and the mobile application does not use any Speech-Recognizer or Text-To-Speech. This is possible because the UI has been made accessible, in this way visually impaired users can take advantage of the native screen reader of the devices.

## 3. Design Issues

In this section, we present the main design issues that emerged while designing the pervasive system (i.e., mobile app and infrastructure), considering our specific case study: providing the University of Bologna buildings with an accessible wayfinding system to support students (including students with disabilities), tourists, and visitors.

The system has been designed considering different constraints, such as: (i) the need to use it in buildings with different configurations and layouts, including new buildings and historical ones; (ii) the possibility to scale the system deployment to the whole University of Bologna facilities (covering a critical number of buildings in five cities); (iii) the buildings can become really crowded (impacting the accuracy of the sensors ); (iv) information can be also related to historical facts and artifacts, related to the University of Bologna’s long history.

### 3.1. Localization Techniques

Different indoor localization techniques, based on the RSSI, have been proposed in the literature. They can be employed with any technology which provides a signal (e.g., Wi-Fi and BLE beacons). In this Section, we present the four main ones. Each of them has its own strengths and weakness, here evaluated to understand the most suitable technique for our case study.

The most intuitive technique is certainly the proximity [44]. It allows one to locate a device that is within the radius of at least one beacon. The user’s (device) position is approximated to one of the beacons. In the case of multiple signals received by the smart device, only the strongest one is considered. Since a user can be located in correspondence of at most one beacon, there is no need to deploy beacons densely, indeed this situation has to be avoided. The main limitation of this technique is surely the location accuracy while the advantage consists in the limited number of beacons required.

Another intuitive technique is lateration, which is a variation of triangulation that employs distances rather than angle measurements [45]. The position of the device is estimated by measuring the distance between the receiver and three or more emitters. The distance can be measured using the RSSI or other measures, such as Time Of Arrival, Time Difference Of Arrival, and Roundtrip Time Of Flight. The combination of two measures usually guarantees more accurate results [44]. This technique assumes that signals are constant over time to be able to accurately locate users reached by at least three signals. Given that beacon signals are very unstable, the performance of this technique easily degrades. Moreover, even if in optimal conditions, to be able to cover every POInt with at least three signals, it is often necessary to set the beacons on high emitting power, causing extensive battery consumption, or to place a higher number of devices.

A more sophisticated technique is fingerprinting [46]. It requires an offline phase and an online one [44]. The offline phase consists of the collection of signals received in each different position of the building. In the online phase, instead, users are localized comparing the signals received by them to the ones collected in the offline phase. Users are located in the position that has the most similar signals to the ones currently received. This approach promises excellent performance thanks to the offline phase performed on-site, but it also suffers from multiple problems [45]. Firstly, it requires the deployment of a dense grid of beacons. Furthermore, as already explained, the beacon signals are not constant over time making the comparison complex. However, the major drawback is that a change in the beacons positioning requires a brand-new off-line phase, which is a time-consuming activity.

Finally, there is Pedestrian Dead Reckoning that can be used only in combination with one of the previous techniques [47]. It is a relative navigation technique that determines the user’s position, starting from a known position and then adding successive position displacements estimated through measurements of distance, speed and direction detected (or supposed) by the smartphone’s sensors.

A precise comparison among these techniques is hard to carry on. An intuitive approach could be considering the accuracy, in terms of meters, of such algorithms. Each solution proposed in the scientific literature is evaluated with different settings, and using various metrics. Hence, comparing the precision of the different techniques becomes nearly impossible.

**Figure 1:**
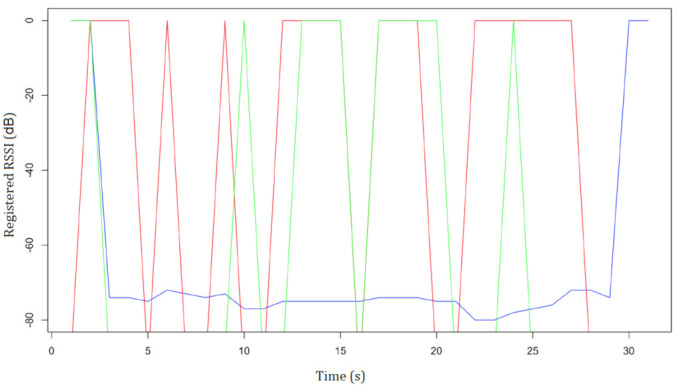
Beacons’ RSSI: beacon1 (**red**), beacon2 (**green**), and beacon3 (**blue**).

In addition, different variables significantly influence the precision of such algorithms, including the physical characteristics of the building, the presence of other signals in the same band, the number of people nearby, and the technical features of the receiving device. All the aforementioned factors lead to very unstable, and consequently unreliable, signals [48]. This can be also observed by the experimental tests conducted with three beacons (same model and same transmission power), deployed in a simple setting, without altering the overall environmental conditions, whose results are reported in Figure 1. As shown, the signals of the beacon1 and beacon2 go up and down, while the signal of the beacon3 has almost the same RSSI.

In order to alleviate the effects of the unreliability of the beacons’ RSSI, some specific measures have been proposed. In particular, such measures aim to eliminate irregularities in measurements employing statistics-based filters, thus using the data they elaborate rather than the actual measurements for calculating the position. Different solutions have been adopted for the lateration technique. Murata et al. [49] employed the particle filter to process the data and combine them with the ones of other sensors integrated into the smartphone. Another variant combines the particle filter with the n-point trilateration [50]. Finally, Chai et al. exploited the Kalman filter [51]. With regard to the fingerprinting technique, Lu et al. combined the k-nearest neighbor with the moving average filter [52] while Sun et al. [53] combined it with a system able to recognize motion patterns previously collected. Instead, the proximity technique is the one that suffers less from the beacons unreliability. In fact, it is sufficient to arrange and set up the beacons so that their signals do not overlap.

Among all the main localization techniques based on beacon technology and after conducting some tests in different buildings, we decided to employ the proximity technique. Despite it is theoretically the least accurate localization technique, it proved to be sufficiently precise for our case study. Take as an example blind users, as the target audience that, indeed, requires more careful thought considering the provision of turn-by-turn navigation. AlmaWhere is designed to help them on the way to the desired destination inside a building. It is not intended to replace other supports, such as a white cane or a guide dog, which are however necessary to avoid obstacles. Moreover, proximity is the most scalable and maintainable solution. It requires neither a dense grid of beacons nor an off-line phase. In this way, it is easier to map a new building inside the system (i.e., deploy the beacons based on the defined POIs) and/or modify the beacons deployment in any of them.

### 3.2. Universal and Inclusive Design vs. Design for a Specific Need

The most suitable design process for obtaining an effectively usable software artifact (as well as a usable UI) is still a debate matter by the experts in the field, in particular when users with special needs are involved in the target audience [54]. In [55], the authors referred to the design as “the identification, discussion and resolution of trade-offs”, where “trade-offs” are namely situations where it is necessary to renounce to something with the aim of earning something else [42,56]. With specific regards to a UI design, such trade-offs are related to the purposes of the software artifact and of its interface and to the constraints and limitations, that could impose to choose among those purposes. This is why a design problem does not have a unique correct solution or an only right answer, in fact, the solution or answer to a design requirement is strongly dependent on the values, the interests, and the needs of the involved stakeholders and users [57].

Dealing with target users groups that include users with special needs, we have to take into account both accessibility, which could be intended as the goal or as an attribute of the interface, and a proper design, that is a method or the process/path that has to be walked so as to reach the accessibility goal [58]. In literature, two totally different design processes can be applied: a universal and/or inclusive design or a design for a specific need [59]. In the latter, the designers take into account only a specific kind of users and their needs, and the solution to the design requirement meets only such needs, without being suitable and usable for other users with different preferences [60]. From the users’ point of view, this could be the best approach, equipping them with the most suitable and tailored UI, designed with the aim of meeting their needs. From the developers’ point of view, this could mean the implementation, the management, and the maintenance of different UIs (or of different applications or software artifacts), whenever it is needed to enlarge the target audience [61].

A long discussion is still ongoing related to the former approach, based on the universal and/or inclusive design [62]. Very often, these two terms are considered synonymous and interchangeable. Others proposed definitions for such terms that have unfocused borders, where the differences between them are not so clear [63].

All this said, it is clear that there are no commonly adopted definitions of these terms, nevertheless, we can define universal design as “The design of products and environments to be usable by all people, to the greatest extent possible, without the need for adaptation or specialized design” [64], while inclusive design can be defined as “The design of mainstream products and/or services that are accessible to, and usable by, specific individuals, with specific needs and preferences, and extending this to others, considering the full range of human diversity with respect to ability, language, culture, gender, age, and other forms of human difference” [65]. Another definition of inclusive design is the following one: “`inclusive design’ is not a fixed set of design criteria, but a constantly evolving philosophy. The goal of creating beautiful and functional environments that can be used equally by everyone, irrespective of age, gender, or disability requires that the design process must be constantly expanding to accommodate a diverse range of users, as we develop a greater understanding of their requirements, desires and expectations” [63].

In this context, what is clear is that these definitions are based on a central role played by the user and his/her needs, trying to meet as many users as possible, by applying UI adaptation and personalization techniques and approaches. As reported in [59] the role of specific target users in the design phases is becoming strategic: their active involvement in the definition of the design requirement and in all the following phases of a UI design is proved to provide great benefit to the quality of the final result [66]. Thus, intrigued by this issue, we decided to move a further step forward by involving a developer with a disability (a blind person) in the implementation team, so as to experience the so-called ”design for user empowerment” [67]. This is based on the idea that some of the best UI and the best interaction systems are obtained when there are people with disabilities on the design and development team, contributing to all aspects of the design and implementation, not just as participants in user studies. This issue can be intended as a deeper form of inclusive design (and development), applying inclusion from also a different point of view [68].

Similar considerations can be done taking into account the development approaches of mobile phone applications, dealing with the two most commonly used operating systems: Android and iOS. Designing and developing native applications is more expensive, time-consuming, and less effective than designing and developing only one mobile application for both of them, but it lets better exploit mobile devices elements, sensors, and functionalities [66]. Choosing just one of the operating system is not an option: on the one side, Android is currently the most commonly used one; while, on the other side, iOS is the operating system used by all the users with visual impairments, thanks to the great support they provide in terms of a dedicated screen reader, quality and precision of the gesture recognition on the multi-touch screen, etc [69]. Thus, the best solution, in this sense, seems to be the use of hybrid applications and related development frameworks.

Summing up, in order to create a software artifact for mobile devices, meeting the needs of a wide target audience (including those users with disabilities), the most suitable and effective design process and app development strategy seem to be the inclusive design and the hybrid app development. Hence, those will be the ones that we will apply in our case study.

## 4. System Walkthrough

This Section presents the details about how we designed and deployed the pervasive system, addressing the different design issues above mentioned, in terms of: (i) localization strategy and beacons deployment; (ii) the design process; (iii) system architecture and data model. Finally, we present the application implemented, focusing on the main functions that can be enjoyed.

### 4.1. Localization Module and Beacons Deployment

As already anticipated, we decided to employ proximity as the chosen localization technique. Once implemented it, the right combination between the beacon settings, the parameters of the localization algorithm, and the best beacon deployments must be found. The beacons settings consists in two parameters: Transmission Time and Transmission Power. Low Transmission Time ensures shorter times for localization but implies more power consumption. High Transmission Power allows us to cover a larger area of the building, yet reducing the accuracy of the localization, making overlap likely, and reducing battery life. On the other hand, even if low Transmission Power requires the use of more beacons to map a building, the localization is more precise, the signals overlap are reduced, and the battery life increases.

The proximity algorithm has to detect the user’s position while moving inside the building. It is possible that at a given instant, the smartphone receives the signal from two or more beacons. In such cases, it is important to be able to discriminate the most powerful one (i.e., the closest beacon), ignoring at the same time the other signals. The latter situation could depend not only on overlapping signals but also on spurious readings [70]. To avoid this situation and to make the algorithm more robust, we implemented it using a window of N signals. Within this window, it is considered the closest beacon the one whose signal has been the most powerful at least K times. The choice of N and K is a trade-off between robustness and the position update speed. Larger N and K ensure robustness but imply that the user’s position is updated after receiving at least K signals.

Given the chosen localization technique, there are no constraints on the arrangement of beacons. Essentially, the deployment is mainly driven by the location of: (i) the turning points, (ii) the dead-end corridors, and (iii) the POIs. The turning points must be obviously mapped to be able to indicate to the user the correct way in the presence of several possibilities. The dead-end corridors, instead, allow one to reach any place in the building. Finally, the points of interest must be mapped to notify the user when s/he is nearby one of these.

Among the different brands of beacons available, we chose the Smart Beacon SB16-2 produced by Kontakt.io (https://kontakt.io/, Accessed on 23 February 2020). They are Bluetooth 4.2 compliant and, with seven transmission power levels available, have a range of up to 70 m. Thus, they support both the iBeacon and the Eddystone protocols.

**Figure 2:**
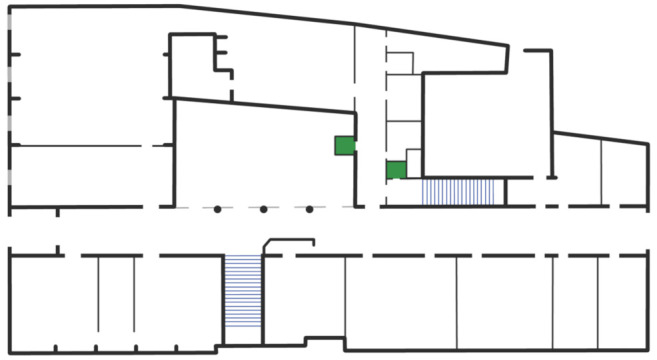
Maps of the ground floor of the building.

The deployment of AlmaWhere took place in three buildings of the University of Bologna, exploiting different contexts and building configurations. To explain the general deployment procedure that we have defined and employed, we describe below the example of the ground floor of the historic Palazzo Riario, headquarters of the Department of History and Cultures, that is depicted in Figure 2.

**Figure 3:**
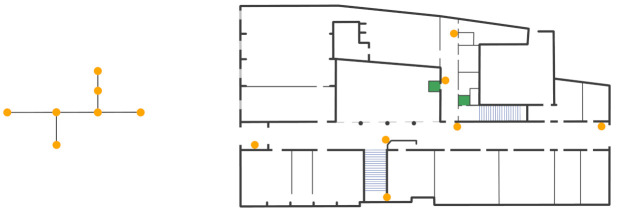
Graph of turning POInts and ends of corridors (**leftmost**) and correspondent beacon deployment (**rightmost**).

Starting from the map of the floor, we have defined a graph G = (V, E) where V are all the turning points and the ends of the corridors and E represents the set of edges that connect such vertices. As turning points are considered also the stairs and the elevators. The resulting graph of the ground floor is portrayed in the leftmost part of Figure 3 while in the rightmost part is reported the correspondent beacons deployment. This is the minimum quantity of beacons needed to develop a navigation system.

**Figure 4:**
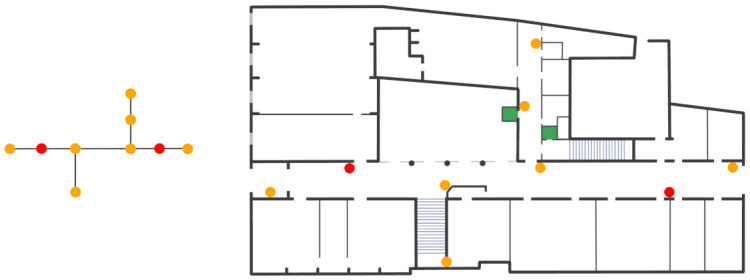
Complete graph (**leftmost**) and correspondent beacon deployment (**rightmost**).

The second part of the procedure consists of associating the POIs to the beacons in the graph. In our system, each POI is associated to one and only one beacon. Instead, a beacon can be associated to zero or N POIs. All the POIs, that are within the range of a beacon, are simply associated to it. However, it is possible that some POIs are not associated to any beacon. In these cases, new beacons have to be introduced so that, in the end, each POI is associated to a beacon. Obviously, the graph changes accordingly, with a new node for each new beacon. The updated graph of the ground floor is reported in the leftmost part of Figure 4. The red points are the nodes added to be able to map some POIs that were not associated to any beacon. In the rightmost part, instead, there is the corresponding beacons deployment.

As far as height is concerned, it must be far enough away from the floor to avoid unexpected behavior due to ground reflection. Furthermore, sufficient ground clearance ensures adequate signal propagation even in the presence of many people. Since our testing building (i.e., Palazzo Riario) has very high ceilings, circa 7 m, we decided to place the beacons at a height of about 2.5 m.

Once the beacons have been deployed, we performed several tests to understand the best combination of all the parameters described above. In the end, we decided to employ the Transmission Time of 300 ms, the Transmission Power set to −20 dBm (i.e., a range of 4 m) while N (the window size) and K (the minimum number of signals required within the window to change the closest beacon) were respectively equal to three and two.

### 4.2. Our Design Process

We focused our design process on the inclusion of target users, driving it into an inclusive dimension. The system has been specifically designed for university communities, with particular attention to students and in particular to students with disabilities. Among them, the system aims to meet the needs of students with visual and/or mobility impairments. This means that it has to be designed so as to bring together two aspects of accessibility: indoor accessibility (in terms of accessibility of the building, by taking into account architectural barriers and facilities) and e-accessibility (in terms of digital accessibility of the system, by taking into account the accessibility of the user interface and of the interaction mechanisms) [71].

Beyond these target groups, other users can benefit from the system, according to the so-called “curb cut effect” [6]: university professors, researchers, and staff (as university communities), tourists interested in the historical buildings within the campus, hosting university offices and venues, as well as people who visit the campus who do not know the university buildings and areas.

With the aim of reaching the result of an effective and efficient system, we have adopted an inclusive design approach, by actively involving some target users in the design process. In particular, we have involved students with visual impairments. Moreover, a blind computer science student has been involved in the development team, so as to better implement and evaluate an accessible user interface and accessible interaction mechanisms, as well as a better definition of indoor beacon positioning. As already stated in Section 3.2, the involvement of a developer with a disability in the development team gave us the chance of applying the so-called ”design for user empowerment” [67], and it has been motivated by the consideration that this is the deepest form of inclusive design and development, since the involvement of persons with disabilities in the development team can significantly contribute in improving all the aspect of the UI and of the interaction flows and mechanisms [68].

Taking into account this latter issue, the involvement of target users in the design phase has resulted in the decision of using the proximity technique: the main reason is that this produces a better balance between the number of beacons needed to equip the building with a wayfinding and navigation system and the fact that people with visual impairments will not give up assistive technologies and devices they use every day while moving independently (such as white canes or guide dogs). Thus, they showed that our system would be used as an additional support [72], without requiring a really accurate positioning and navigation system, but robust enough to use a system that could show some errors in the turning points. Moreover, this design approach has brought also the idea of exploiting assistive technologies already installed in the user’s mobile device, together with the most commonly used Text-To-Speech feature. This means that a blind student can interact with our system by means of a mobile app with his/her mobile phone screen reader (keeping his/her preferred configurations and settings).

### 4.3. System Architecture and Data Model

The system architecture has not undergone major changes compared to previous works [69,73]. The data about the buildings are stored in a database on the server. A web application, implemented using the PHP framework Symfony (https://symfony.com/, Accessed on 23 February 2020), allows us to manipulate them, performing CRUD operations on all the system entities (e.g., buildings, beacons, vertex, edges, …). Furthermore, the server will expose specific APIs to allow mobile applications to retrieve the data. Once downloaded the data from the server, the mobile application will interact with the beacons to determine the user’s position, allowing the user to enjoy the various features. The retrieved data will be used to build a graph and Dijkstra’s algorithm will be exploited to find the shortest path between the users’ current position and the selected destination. In order to improve the overall scalability of the system, the wayfinding algorithm will be implemented client-side. This solution brings several advantages including less computational load on the server, less bandwidth consumption, and above all a faster recalculation of the path which can significantly affect the user experience.

**Figure 5:**
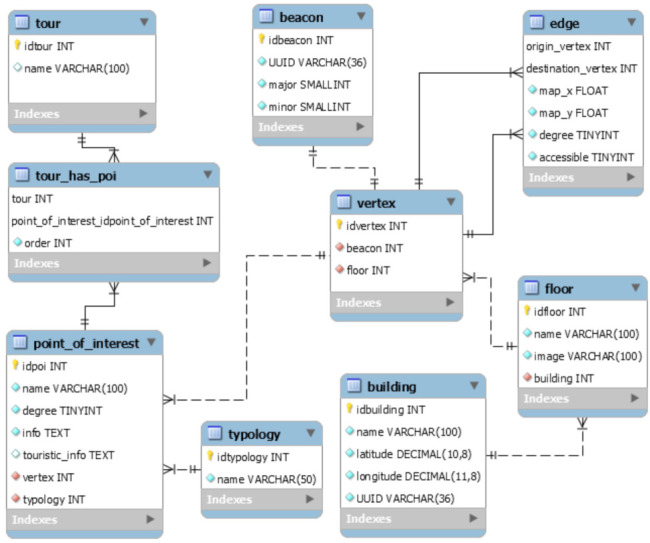
AlmaWhere Data Model.

In Figure 5, we reported the entity-relationship model of AlmaWhere. Each building is characterized by the name, geographical position, and UUID. The building has one or more floors, each of which contains the image of the map that will be shown when the user is on the specific floor. With regard to the navigation graph, there are two main entities: vertexes and edges. Even if the vertexes coincide with the beacons, we kept them in separate tables as the positioning of the beacons inside the building may be subject to changes based on maintenance operations. The beacon contains the UUID, the major, and the minor while no additional information is associated to the vertexes. The edge, instead, connects two vertexes. Even if each edge is bi-directional, it is stored on the database considering one single direction, with the aim of saving space. Together with the origin and the destination nodes, four other pieces of information are saved: the coordinates (x,y) of the map portion relative to such edge, the degree of difference between the origin and the destination node, which is used to indicate in which direction the user must move (i.e., go ahead/back, turn right/left), and a boolean specifying that such edge is accessible to people with mobility impairments or not. As already stated, zero or N POIs can be associated to a vertex. A POI has the following attributes: name, degree, info, touristic info, and typology. Finally, there are tours, that essentially include ordered lists of POIs.

### 4.4. Our System at a Glance

In this section, we detail the functionalities of our mobile application. We implemented it with the React Native framework (https://facebook.github.io/react-native/, Accessed on 23 February 2020), which allowed us to create native apps for Android and iOS using a common Javascript codebase.

#### 4.4.1. Home and Settings

When launched, the app interacts with the server to understand if there is an updated version of the data, and eventually downloads it. Then, it tries to understand the building where the user is located, adopting two different strategies. The first one consists of sensing a beacon and reading its UUID, which is unique for each building. The second one, which is used if the first does not give results, employs GPS to get the current latitude and longitude and compare them with the ones of all buildings. The nearest building is then selected. Anyway, a user can manually select a building.

**Figure 6:**
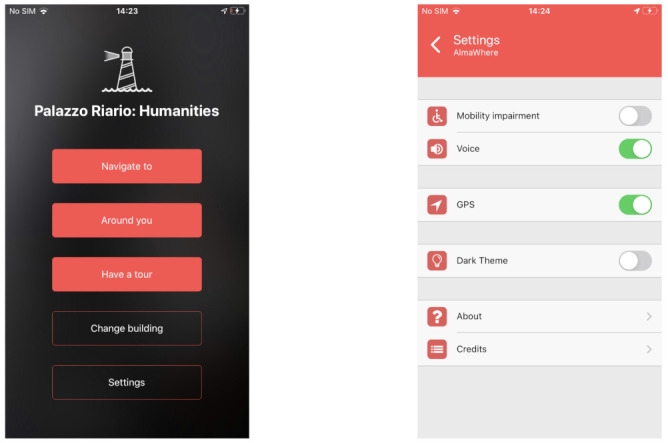
Homepage (**leftmost**) and settings (**rightmost**) of the mobile application.

Once the building has been identified, the homepage, reported on the leftmost part of Figure 6, is shown. It is composed of a title, containing the name of the building, and of five buttons: the first three ones are related to the three main functionalities of the app, the fourth one allows us to change the building, while a tap on the last one open the settings, whose screen is depicted in the rightmost part of Figure 6.

In the Settings view, it is possible to specify whether or not the user has mobility impairments (temporary or not). When checked, this option filters out the edges not marked as accessible in the path computation. Instead, there is no option for visually impaired users, since the UI (User Interface) is accessible (i.e., the screen reader can read the displayed text) and there’s no special need in the path computation. The other options allow us to enable the Text-To-Speech, the GPS, and the dark theme. Finally, there are two information panels with a description of the app and the credits.

#### 4.4.2. Navigate to

**Figure 7:**
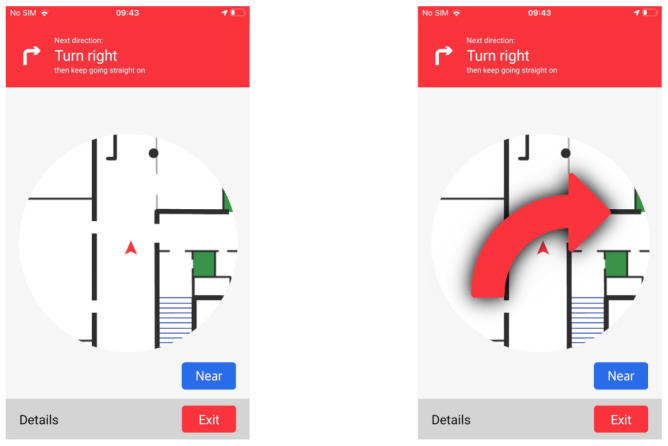
Navigation UI: general view of the map (**left**) and turn signal (**right**).

This functionality takes care of leading the user from his/her current position to a specific destination, chosen from the list of POIs of the building. First of all, the graph is created using the information about the building. Then, the route is computed with Dijkstra’s algorithm, using the library *node-dijkstra* (https://github.com/albertorestifo/node-dijkstra, Accessed on 23 February 2020). Once the path is computed, the navigation starts, showing the Navigation UI, reported in Figure 7. It is divided into three sections. The top one contains the next indication to follow. In the center, there is always the portion of the map relative to the edge that the user must travel. Its orientation varies according to the device compass. If there are POIs associated to it, the button “Near” is shown. The bottom bar, instead, contains the buttons “Details” and “Exit”.

The indications are provided at the beginning of the navigation and whenever the nearest beacon changes. They are based on the device orientation and on the degree of the edge that the user must travel. In the example of Figure 7, the app has already shown the message to go straight ahead and it now shows the next indication (i.e., turn right) on the top bar and the map relative to the current edge. When the app senses the beacon associated to the turning POInt, the next indication becomes the current one, and it appears over the map for a predefined time interval (currently set to 3 seconds), as shown in the rightmost part of Figure 7. Then, it disappears and the map is updated, together with the next indications in the top bar.

During the navigation, if the app detects a beacon, that is not part of the established route, it recomputes a new route using the last sensed beacon as the starting point. All route computations take place on the device and not on the server to ensure a rapid reaction and full functionality even in the absence of connectivity.

**Figure 8:**
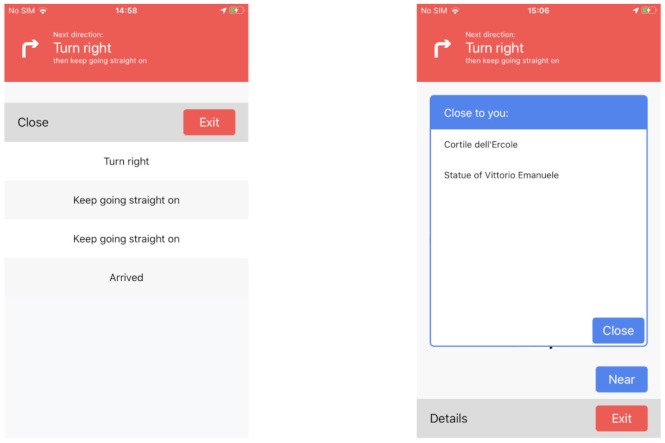
Navigation UI: details view (**left**) and near view (**right**).

At any time of travel, the user can tap on one of the three buttons. The bottom “Exit” simply interrupts the navigation, showing the homepage. The “Details” one shows a sliding panel containing the list of all the indications to reach the destination, also depicted in the leftmost part of Figure 8. “Near”, instead, opens a pop-up with the list of the nearby POIs (i.e., the ones associated to the last sensed beacon). The user can view any cultural information of a POI by clicking on its name, without interrupting the navigation that continues in the background.

Finally, when the destination is reached, a final indication is computed taking into account the orientation of the device and the one of the POI.

#### 4.4.3. Around You

This feature allows one, once a beacon is sensed, to get the list of the nearby POIs. It has a double aim. On the accessibility side, it allows blind people to build a mind map, becoming familiar with the buildings the first few times s/he visits it. On the museum side, instead, it helps users who see particular POIs in getting more cultural/historical information.

**Figure 9:**
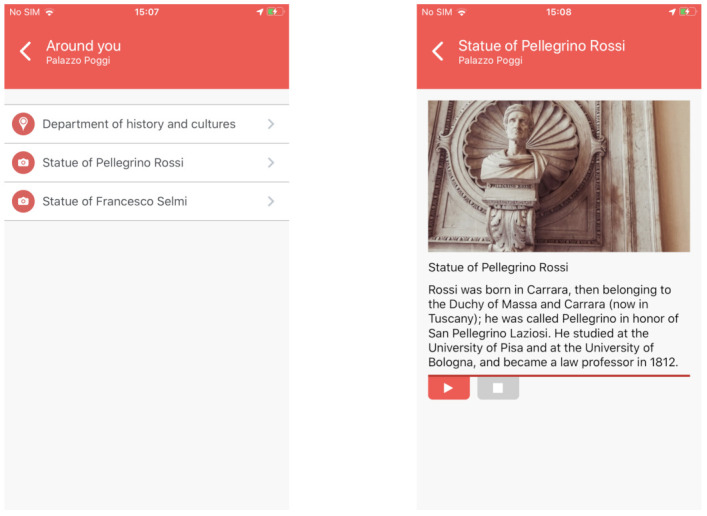
Around you UI: List of POIs (**left**) and POI Details (**right**).

The list of nearby POIs, which are flanked by an icon that indicates whether or not they have a historical value, is reported on the leftmost part of Figure 9 while in the rightmost one, it is depicted the details of a particular POI belonging to the historical Palazzo Poggi (the headquarters of the University of Bologna and of the rector of the university), that is the Pellegrino Rossi statue. The details consist of a photo, a title, and a description, that can be vocalized by the Text-To-Speech.

#### 4.4.4. Have a Tour

**Figure 10:**
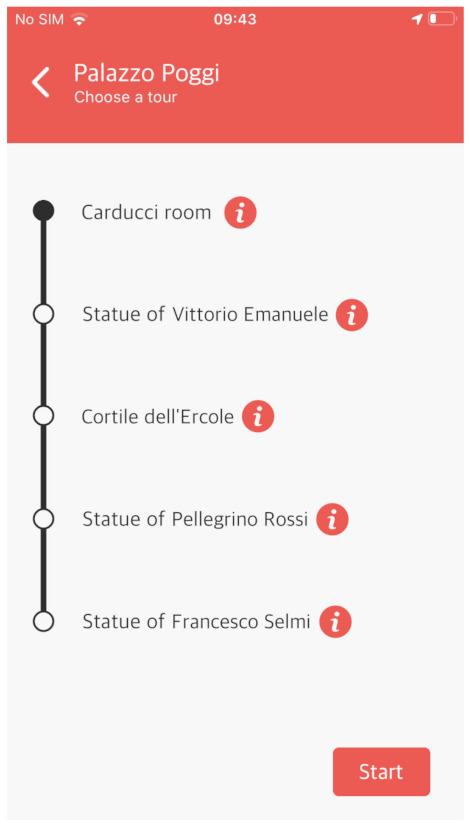
Tour UI.

The last feature of AlmaWhere is “Have a Tour”. It does not allow the user to choose a single destination but it guides him/her across a predefined list of POIs that presents some peculiarities from the cultural and artistic Points of view. The idea is to propose a set of theme tours, that allow users to admire some POIs like statues and paintings, present in the building. An example is reported in Figure 10 where the screenshot is reporting the list of POIs included in such a tour.

The indications are provided as in normal navigation, but upon reaching each POI, its descriptive card is shown. When the user has finished reading the description, s/he can continue the tour by tapping the “Continue tour” button.

If there are no tours available in a given building, the button “Have a tour” is not shown on the homepage.

## 5. Field Studies

To assess the efficacy of our approach, such as the accessibility and opportunities of use of our system, we conducted three different evaluation sessions in natural settings (i.e., in the University premises). Each field study targeted a specific location (natural setting) and a specific target group, following a purposive sampling (i.e., grouping participants according to preselected criteria). The three evaluations were set up in different scenarios, as briefly presented in Table 1, and described in detail in the next subsections.

### 5.1. Methodology

All the sessions were organized following the same protocol. Firstly, we set up the infrastructure, locating the beacons considering the more strategic deployment, following the process described in Section 4.1, on the basis of the building structures and the declared POIs. The experimenters performed some tests to confirm that the infrastructure was set up in the correct way. After, we received the participants. We firstly made an introductory description of the purpose of the research, informing them about what is expected of a research participant, including the amount of time likely to be required for participation and the fact that the participation is voluntary and that one can withdraw at any time with no negative repercussions. Then, we performed the evaluation session, one user at a time. We provided the user with an iPhone 8 or with a Redmi Note 7 (with Android 9) where AlmaWhere was installed, to avoid asking users to install new software on their personal smartphones. We provided users with the possibility to choose Android or iOS to avoid issues due to using an unfamiliar operating system.

Each evaluation session was performed with two researchers, one to assist the user during the wayfinding activity, and the other one, following the session a couple of steps back, collecting data and annotating feedback, reactions, and the precision of the provided step-by-step navigation info. After the session, another researcher performed a structured interview to collect both qualitative and quantitative data, recording the participants for future coding and analysis (after providing them the information on the processing of the collected data and obtaining their oral—considering blind users—or written informed consent, accordingly with the General Data Protection Regulation). The informed consent was approved by the Privacy and Data Protection Office of the University of Bologna, and supervised by the Services for disabled students and students with specific learning disabilities.

The structured interview consists of different predefined questions. In particular, the interview comprised different sections: we started with (a) some questions related to general information (age, gender, etc.), we continue with (b) questions related to their familiarity with apps for indoor/outdoor wayfinding, then, (c) specific questions for each function (i.e., “Navigate to” and “Around you”) were asked, using a five point Likert scale (from “very satisfied” to “very dissatisfied”, including the neutral value), finally, we conclude the interview with (d) an open question to collect general feedback and comments. Each participant went through all the sections, but the questions were slightly different in accordance with the different targets considered, to gather relevant aspects peculiar to each group’s needs.

#### 5.1.1. Evaluation with Students with Disabilities

We organized a session with five students with disabilities (four blind users and one wheelchair user) in the main building of the Department of History and Cultures. Students (two females) were from 19 to 44 years old (mean = 26.4 and sd = 8.8), enrolled in different courses, such as Computer Science, Law, and Arts, Humanities, and Cultural Heritage. We selected such a building since it includes the Tecnolab, a technological laboratory created to welcome students with disabilities, equipping them with different assistive technologies they can try and explore within the lab, together with specialized tutors. The lab has been created in collaboration with the Services for disabled students and students with specific learning disabilities of the University of Bologna (in the Italian language: “ABIS—Settore Diritto allo studio—Ufficio Servizi per gli studenti con disabilità e con DSA”). We asked participants to: 1. start from the main entrance and to look for the closer toilet (for females/males/wheelchair users); 2. once at the toilet, check for nearby POIs; 3. from the toilet, reach the library at the 1 floor (using the accessible elevator or the stairs, on the basis of the user’s needs). All the students were familiar only with the Tecnolab premises and not with the building itself.

#### 5.1.2. Evaluation with Visitors and Tourists

We randomly engaged five users who voluntarily accepted to participate in the study: we simply approached them close to the main entrance/exit of Palazzo Poggi. In particular, we involved two tourists from Brazil (mother, 54 years old and son, 19 years old) who were there to visit the Poggi Museum, and three students, from 20 to 24 years old (two females) there to visit, for the first time, the library and the famous classroom dedicated to Giosué Carducci, in which the poet gave lectures on the Italian language and literature for 40 years.

In this case, we asked users to: 1. reach the Pellegrino Rossi statue, then, 2. retrieve information about the statue, and, finally, 3. perform a short tour (selecting the only one available on the prototype).

#### 5.1.3. Evaluation with Campus Students

We involved eight students (one female) attending classes at the Campus of Cesena. These students were from 23- to 26-year-old (mean = 23.6, sd = 1.2; one female). One of the participants was experiencing a temporary mobility impairment (broken leg). In this evaluation, we only asked users to reach a particular not well-known classroom inside the Campus.

### 5.2. Results and Discussion

**Figure 11:**
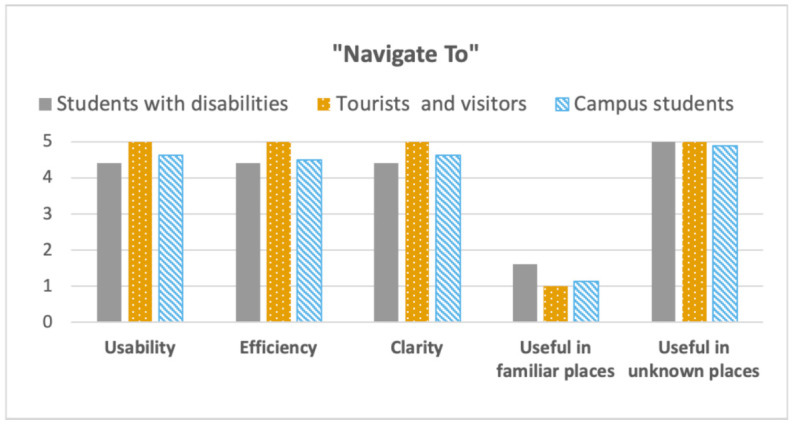
“Navigate To” function: Likert scale average values, comparing the different target users.

**Figure 12:**
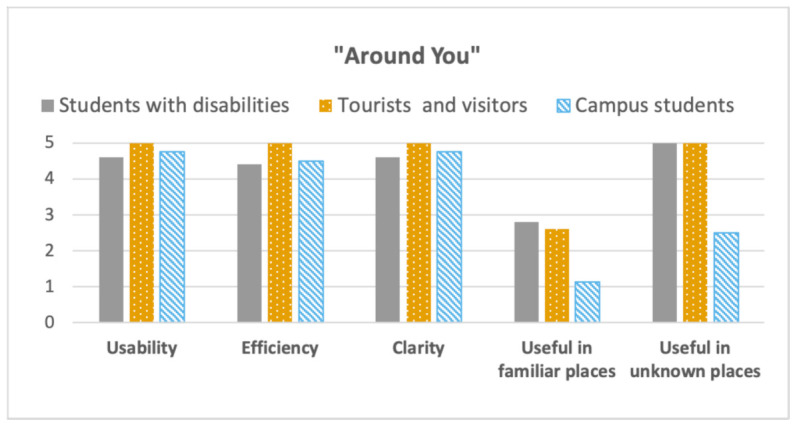
“Around you” function: Likert scale average values, comparing the different target users.

Figure 11 and Figure 12 show the outputs of the questions related to the “Navigate To” and “Around you” functions, considering five dimensions: Usability, Efficiency, Clarity, Useful in familiar places, Useful in unknown places. The outcome showed that, on average, the users found both the functions usable, effectiveness to use, and clear, with values close to 5 (the maximum). Instead, negative values (less than 3) were obtained when we asked about the usefulness of the function in familiar places. As expected, values were particularly negative when considering the “Navigate To” function (less than 2). Values were a bit higher considering the “Around You” function in familiar places, at least considering students with disabilities and tourists and visitors. That can be explained considering the fact that using such a function, it is possible to learn information about points of interest. A point of interest could be a historical artifact (considering visitors and tourists), or a toilet (accessible or not), for females or males (considering users with disabilities), or vending machines for coffee and drinks (considering users with visual impairments). Nonetheless, once one became familiar with a place, this function also lost interest. Accordingly, students with visual impairments mostly picked the neutral value (3) to rank this function. The reason is that they loved the function and they think it is incredibly useful in unknown places, but, once one is getting used to a place, s/he should not need it anymore. Focusing on visitors, one commented: “I think the function can be really interesting the first time you use it (independently if the place is familiar or not). Then, the statue, for example, will be always the same. This is why I ranked 1”. Contrariwise, a visitor who assigned four to the function, claimed: “Also if you come here [Palazzo Poggi] often, I don’t think this statue will attract your attention, so you need a system that can facilitate this and let people learn about the story of the represented person”. Considering the results gathered in the Cesena Campus, almost all students assigned one to the “Around You” function in familiar places. Checking the data, it is possible to observe that they assigned a low score also when using such a function in unknown places. The motivation can be found considering that the Cesena Campus is a completely new building, with no artwork or historical artifacts. Nonetheless, we are planning to make the function more engaging, including information about the campus, such as lesson schedules, professors’ reception hours. A very encouraging result is that, in all the field studies, the “Navigation To” function obtained the maximum score (5) when considering unknown places.

Focusing on the users with disabilities, different insights emerged. On the one hand, users with mobility impairments really appreciated the system, being surprised by the calculated path including the accessibility facilities (e.g., accessible elevators), avoiding barriers (e.g., stairs). On the other hand, three out of four blind users were expecting a more precise system in terms of step-by-step navigation, but, despite that, they recognized the relevance of such a system: none of the participants ever used a similar system for indoor navigation, and they were positively impressed. One limitation that emerged during the study was related to the smartphone orientation. In fact, the application exploits also the compass to provide information to the user, but visually impaired people often prefer to keep the phone in a bag (using headphones to control it) or keep it in a landscape position, to have the micro close to the ear. To solve this issue, we decided to allow users to disable the compass and rely only on the beacons mapping.

Tourists and visitors really liked the “Around You” and the “Tour” functions. One tourist said: “The Around you function is super interesting and, in this building [Palazzo Poggi] full of historical facts, it can become a very important tool, a kind of virtual guide, that allow you to explore every corner of this place”. At the same way, they all appreciate the “Tour” function. One tourist claimed: “When I enter this building, I felt very confused. It is huge and very complicated to navigate. Having an app that can provide you step-by-step navigation following touristic tours can really enhance the visitor’s experience of such a beautiful and historical place”.

Students in Cesena understand the idea behind the application and, in general, appreciate it. Nonetheless, most of them suggested to alleviate the loss of interest in using the app in familiar places by including some other information related to the University premises and the user’s position, such as the class schedule. Accordingly, one reported: “The app is interesting and can provide relevant information to reach a Professor’s office or a lab for the first time, but then, it has to provide me something more, something more dynamic, such as the lessons’ timetable, otherwise I stop using it”. Moreover, as already anticipated, students (without disabilities) in the new Cesena campus also didn’t completely understand the potential of the “Around you” function, due to the absence of historical artifacts or facts and the possibility to visualize the point of interests while facing them. This is an interesting result that can be taken into account in refining the app. In fact, the app can simply allow each user to personalize the app, choosing which functions to enable and when.

Due to the limited number of participants who tested the system, we can not provide the statistical significance of the collected quantitative data. Nonetheless, we can state that the general feeling was very positive and participants enjoyed using it.

## 6. Conclusions and Future Work

In this paper, we present how a mobile application, together with an IoT infrastructure (exploiting BLE beacons) can be exploited to locate a user inside an indoor environment and to provide: (i). wayfinding real-time instructions; ii. location-based information about the mapped points of interest; (ii). predefined tours, including different points of interest. The system has been designed to be accessible, following an inclusive design approach, in order to address the needs and preferences of different target users, including blind users, mobility-impaired users, and visitors/tourists. Three evaluation tests have been carried out to assess the enjoyability and accuracy of the system, showing that the system has a good trade-off between the facility of use and the accuracy of the user’s positioning. Moreover, thanks to its low-cost and easy deployment process, it can be scalable and highly adaptable to different building settings and layouts, addressing our initial constraints.

Reflecting on the lessons learned during the design and implementation of our case study, we can confirm that following an inclusive design approach was strategic in achieving positive results, in terms of usability, efficiency, and clarity of the system, for all the users involved, confirming previous findings [56]. In fact, we defined the requirements considering different target groups (users with disability, tourists and visitors, and students), with specific needs and preferences, and we design a system able to satisfy the different needs, keeping accessibility in mind and taking advantage of the curb-cut effect [6]. Moreover, having the possibility to put into practice the “design for user empowerment” approach, having a blind user in the development team, prevent our system to include issues related to the accessibility and usability of the system, especially for low vision and blind people [67].

As future work, we just started to investigate the use of conversational user interfaces (such as the Amazon Alexa) in combination with our system, to understand if it can be useful in assisting users during wayfinding tasks.

## Figures and Tables

**Table 1:** Details about the three evaluation sessions.

Location	Description	Number of Users	Target
Palazzo Riario	The headquarters of the Department of History and Cultures, Bologna. It includes the *Tecnolab*, a technological laboratory equipped with different assistive technologies, created to welcome students with disabilities.	5	Students with disabilities
Palazzo Poggi	Built in 1549, it is the headquarters of the University of Bologna and of the rector of the university. It also includes a museum, a library, some classrooms, and several administrative offices.	5	Visitors and Tourists
Cesena Campus	It is one of the new Multicampus structures of the University of Bologna build in the city of Cesena. The new campus building hosts different degree courses and department facilities.	8	Students

## Data Availability

No applicable.

## Abbreviations

The following abbreviations are used in this manuscript:

PoI	Points of Interest
BLE	Bluetooth Low Energy
GPS	Global Positioning System
RSSI	Received Signal Strength Indicator
RFID	Radio-Frequency IDentification
IoT	Internet of Things
UUID	Universally Unique IDentifier
UI	User Interface
